# CEO Empowering Leadership and Corporate Entrepreneurship: The Roles of TMT Information Elaboration and Environmental Dynamism

**DOI:** 10.3389/fpsyg.2021.671232

**Published:** 2021-07-15

**Authors:** Zhengfei Li, Huangen Chen, Qiuying Ma, Haibo Li

**Affiliations:** ^1^School of Economics, Shandong University, Jinan, China; ^2^School of Business Administration, Faculty of Business Administration, Southwestern University of Finance and Economics, Chengdu, China; ^3^Department of Economics, University of Warwick, Coventry, United Kingdom; ^4^Institute of Science and Technology for Development of Shandong, Qilu University of Technology (Shandong Academy of Sciences), Jinan, China

**Keywords:** empowering leadership, information elaboration, corporate entrepreneurship, environmental dynamism, top management teams

## Abstract

In this paper we investigate the relationship between chief executive officer (CEO) empowering leadership and corporate entrepreneurship. In addition, the mediating role of information elaboration in top management teams (TMTs) and the moderating role of environmental dynamism are examined. Drawing on the information exchange/sharing perspective, we hypothesize that CEO empowering leadership has a positive effect on corporate entrepreneurship, and TMT information elaboration mediates the relationship above. Furthermore, we find that environmental dynamism positively moderates the relationship between empowering leadership and information elaboration, and negatively moderates the relationship between information elaboration and corporate entrepreneurship. Data from a sample of Chinese firms provide empirical evidence in support of these hypotheses.

## Introduction

In order to respond effectively to a changing work environment, a management style known as empowering leadership has proved favorable for promoting various positive outcomes, such as enhancing employees’ perceptions of autonomy, creativity, knowledge sharing, efficiency, and firm performance (Srivastava et al., 2006; Zhang and Bartol, 2010; Carmeli et al., 2011; Cooper et al., 2019). Despite this positive implementation, extant research on the effects of empowering leadership has focused mainly on low-to-middle level managers (e.g., Srivastava et al., 2006; Vecchio et al., 2010). Based on the upper echelons theory, managerial characteristics influence corporate strategy choices. It is crucial that it is acknowledged that such effects may also be applicable to chief executive officers (CEOs) or other executive leaders (Vera and Crossan, 2004). In addition, as firms tend to treat innovation as one critical indicator of performance assessment, exploring the antecedents of corporate entrepreneurship is of great practical significance (Jung et al., 2008). In this paper, the main research question is whether CEO empowering leadership has a positive effect on corporate entrepreneurship, and whether top management team (TMT) information elaboration mediates this relationship. The paper also studies whether the environmental dynamism moderates the relationship between empowering leadership and information elaboration, and the relationship between information elaboration and corporate entrepreneurship.

Corporate entrepreneurship can be defined as a process by which an individual or team of individuals, in association with an existing organization, creates a new and competitive organization or instigates renewal or innovation within the existing organization (Sharma and Chrisman, 1999). While innovation is the core of corporate entrepreneurship, it is also the responsibility of every top manager (Darnall and Edwards, 2006). Thus, this study examines the effects of CEO empowering leadership on corporate entrepreneurship at the firm level. Moreover, in the emerging Chinese economy, the power distance between leaders and subordinates is much greater, allowing CEOs to exert a large amount of influence over TMT members (Zhao et al., 2020). Thus, this study regards the work dynamics in China as a useful context for elucidating how CEO empowering leadership may influence corporate entrepreneurship.

In building a model linking empowering leadership and corporate entrepreneurship, we draw on the information/decision-making and upper echelons perspective to posit one internal process mechanism as a mediating variable between empowering leadership and corporate entrepreneurship (Van Knippenberg et al., 2004). According to the information/decision-making perspective, information elaboration in the TMT is a specific factor in this internal process mechanism (Van Knippenberg et al., 2004). Information elaboration at the team level can be defined by the degree to which information is exchanged, shared, and processed by the group’s members (e.g., Van Knippenberg et al., 2004; Yuan and van Knippenberg, 2020). Based on the assumption that the TMT in a given firm operates as a team or group, we believe that research findings on information elaboration in general can also be applied to the TMT context. TMT information elaboration is critical as an internal process mechanism, as it allows TMT members to work together to use their knowledge resources via information elaboration (Marks et al., 2001; Kearney et al., 2009). Assuming that TMT information elaboration is a firm’s entrepreneurial source (Penrose, 1959), scholars tend to conclude that firms should employ empowering leadership to strengthen their TMT information elaboration (e.g., Latham et al., 1994). Moreover, according to previous studies (e.g., Dess et al., 2003), corporate entrepreneurship can be improved through information exchange across multiple management levels. This type of information exchange is considered an important dimension of successful organizational learning, which has not been sufficiently tested in previous empirical studies and is thus the focus of our current study. In this sense, the influence of a firm’s empowering leadership on corporate entrepreneurship may be due to the mediating effect of TMT information elaboration. However, the existing research focuses on the mechanism between empowering leadership and corporate entrepreneurship and ignores the TMT information elaboration. Thus, this study seeks to fill the research gap by investigating the mediating role of TMT information elaboration in empowering the leadership–corporate entrepreneurship relationship.

Chief executive officers often confront various levels of uncertainty and complexity in the business environment, which requires them to interact dynamically with their subordinates (Hambrick, 1994). Whatever the environment, CEOs have an obligatory responsibility to lead their organizations and motivate their subordinates to confront challenges and move through difficulties that arise. As this ability to lead effectively significantly determines an organization’s eventual performance, it is also of theoretical importance to understand to what extent empowering CEOs are able to influence their subordinates and what factors facilitate and inhibit the relationship. Prior research shows that environmental dynamism may moderate leadership effectiveness (e.g.,Waldman et al., 2001). Therefore, another purpose of this study is to examine the moderating role of environmental dynamism in the relationship between empowering leadership and corporate entrepreneurship.

In addition, perceived environmental dynamism is often considered to be an important contextual variable that moderates the relationship between a firm’s internal resources and its performance (e.g., Waldman et al., 2001; Zhang et al., 2020). If we consider TMT information as an important firm resource, it is of considerable research interest to test whether environmental dynamism can also influence the relationship between information elaboration and corporate entrepreneurship. Therefore, an additional motivation for this study is a desire to examine the moderating effect of environmental dynamism on the information elaboration-corporate entrepreneurship relationship.

The rest of this paper proceeds as follows. First, we provide a review of empowering leadership and corporate entrepreneurship, and then present a conceptual model that helps to bridge the gap in the empowering leadership–corporate entrepreneurship relationship. Second, we examine and discuss the mechanisms underlying the relationship between CEO empowering leadership and corporate entrepreneurship. Third, we describe the research design and report the results of the hypotheses testing. Finally, we conclude the paper with a discussion on the theoretical and practical implications of our findings, the limitations of the study, and directions for future research.

## Theoretical Background and Hypotheses

### CEO Empowering Leadership and Corporate Entrepreneurship

Defined as a process by which an individual or a team of individuals creates a new and competitive organization or instigates renewal or innovation within the existing organization (Miller, 1983; Sharma and Chrisman, 1999), the concept of corporate entrepreneurship consists of three dimensions—venturing, innovation, and self-renewal (Miller, 1983; Zahra and Covin, 1993)— emphasizing the company’s pursuit of opportunities through innovation, creating new business, or producing new products (Stevenson and Jarillo, 1990; Schmelter et al., 2010; Cabral et al., 2021). Corporate entrepreneurship requires firms to initiate innovative actions continually and proactively, while employees are expected to act and think in ways that are entrepreneurial (Kanter and Richardson, 1991).

Along with its antecedents, there is a growing body of literature focusing on the factors that facilitate corporate entrepreneurship. These factors include the firm’s leadership and structure (e.g., Zahra, 1995; Mahmood and Arslan, 2020), resource availability (e.g., Nohria and Gulati, 1996), strategic management (e.g., Miller, 1983; Barringer and Bluedorn, 1999), and human resource management practices (e.g., Schmelter et al., 2010). Among these myriad factors, many researchers have identified leadership behavior as one of the most important factors affecting corporate entrepreneurship (Amabile, 1998; Jung, 2001; Jung et al., 2008; Tang et al., 2012; Imran and Masood, 2018). It is suggested that CEOs have considerable influence over organizational outcomes, including corporate entrepreneurship (Wang et al., 2011). The CEO, CFO, owner-manager, entrepreneurs, and other managers are responsible for making decisions, particularly in small and medium-sized enterprises and venture companies (Sosna et al., 2010). Based on the upper echelons theory, a managers’ characteristics can influence strategic decisions (Hambrick, 2005). However, most of the extant research on corporate entrepreneurship focuses primarily on the external environmental effects, while neglecting the role of top managers (Zampetakis and Moustakis, 2010). Thus, investigating how CEO empowering leadership influences corporate entrepreneurship is of paramount importance. Empowering leadership involves a transfer of power from top management to employee with high autonomy and who are able to take initiative and make decisions about daily activities (Arnold et al., 2000). Consistent with the arguments of previous studies (Bunderson, 2003; Cannella and Holcomb, 2005), we argue that CEO empowering leadership has an important influence on corporate entrepreneurship.

First, empowering CEOs who lead by example exhibit their commitment to their own work and the work of TMT members in order to achieve high levels of team performance. This collective commitment to accomplishing goals encourages team members to contribute their diverse knowledge and skills (Srivastava et al., 2006), thus facilitating employees’ involvement in the innovative process. Second, the coaching behavior of empowering CEOs helps TMT members to become more innovative and capable in performing their work (Arnold et al., 2000). Through the coaching process, the CEOs can enable TMT members to develop innovative thinking and capabilities. In addition, TMT members can enhance their innovative motivations by learning from their CEOs. This, in turn, is beneficial to a firms’ entrepreneurial activities (Finkelstein et al., 1996; Gao et al., 2011). Third, CEO empowerment promotes participative decision making, which encourages TMT members to provide and share knowledge and information (Srivastava et al., 2006) within the TMT. Through such a process, TMT members can expand their knowledge, learn from one another, and acquire new skills, thereby improving their innovative abilities (Latham et al., 1994). Such high-quality synthetic decision making enables TMT members to quickly develop creative and constructive solutions, which in turn fosters the firm’s entrepreneurship. Fourth, empowering CEOs are in favor of sharing information with others. The sharing process promotes the dissemination of information, such as the firm’s mission and vision, so that subordinates are informed about what to do and where to go. Frequent and close communication among team members facilitates an innovative atmosphere that enhances creativity and progress. Finally, empowering CEOs show concern for and take care of their subordinates, creating a sense of ownership and control over the work to be performed among TMT members (Liden and Tewksbury, 1995; Mumford et al., 2002). Under such circumstances, TMT members have greater enthusiasm and flexibility in coping with first-hand information and other important problems that may arise. Hence, firms should be better at exploiting opportunities and enhancing their innovative capabilities.

In addition, numerous empirical studies provide other evidence to support the above arguments. For example, individuals who work in autonomous environments are found to generate more creative ideas (Zhou, 2003). Some scholars (e.g., Jung and Sosik, 2002) argue that empowering leadership can develop individual intrinsic motivation, which facilitates creative endeavors and is an important driver of individual creativity. This then directly contributes to a firm’s overall entrepreneurial process and activities (Amabile, 1998; Zhou, 2003; Jung et al., 2008). Taken together, the above arguments support the enhancement of corporate entrepreneurship through CEO empowering leadership in firms. Therefore, we propose the following hypothesis:

*Hypothesis 1: CEO empowering leadership is positively related to corporate entrepreneurship.*

### The Mediating Role of TMT Information Elaboration

According to (e.g., Chaiken and Trope, 1999), empowering leadership may improve information elaboration because certain elements of such leadership have direct effects on it. Arnold et al. (2000) identify five elements of empowering leadership: leading by example, participative decision-making, coaching, explaining, and showing concern for/interacting with the team. Of these elements, interacting and explaining (i.e., explaining company decisions, goals, rules and/or the leader’s own decisions) seem highly relevant to information elaboration (see the measurement instrument developed by Arnold et al., 2000). All of these elements of leadership primarily serve to improve the information exchange between a leader as an individual and his or her subordinates. In other words, empowering leadership is largely an individual-level measure testing the behavior of a given team leader. It should take place between each pair of members in the TMT and among all members of the team as a whole.

Nevertheless, several researchers, including(e.g., Larson et al., 1998), suggest that there is a positive relationship between empowering leadership and information elaboration. More specifically, an empowering leader provides greater guidance to his or her team members and motivates them to share information and/or ideas with one another. Moreover, if this leader succeeds in building a trusting atmosphere, then his or her empowering leadership is also likely to enhance information sharing among team members. Finally, empowering leaders are also likely to pay greater attention to the development of the information system within their organization, which can also encourage information elaboration.

Taken together, this evidence suggests that information elaboration at the TMT level is likely to be enhanced by empowering leadership at the same level. In other words, as the leader of a TMT, a CEO who empowers leadership is expected to play an important role in promoting information elaboration, which leads us to hypothesize the following.

*Hypothesis 2: CEO empowering leadership is positively related to TMT information elaboration.*

As previously noted, team-level information elaboration is a process by which information is exchanged, shared, and processed among team members (Van Knippenberg et al., 2004; Homan et al., 2007). Different from individual-level variables, such as CEO leadership style, information elaboration has been studied primarily as an ordinary group-level variable rather than at the TMT level. Information elaboration encompasses such types of group behavior as group members complementing one another by openly sharing knowledge and information and group members considering all perspectives in their decision-making (Van Knippenberg et al., 2004).

There have been some studies on the relationship between information elaboration and corporate entrepreneurship (Marks et al., 2000; Wei and Wu, 2013). For instance, Wei and Wu (2013) argue that the elaboration of task-related information reflects the nature of information processing by TMTs, and should have a positive effect on firms’ financial and innovation performance because the sharing of various types of knowledge, skills, and experience among team members is likely to inspire new ideas and knowledge (Van Knippenberg et al., 2004). In addition, access to more comprehensive information can help team members to determine the correct course of action, thereby enhancing the team’s creative ability (Kirkman and Rosen, 1999). What is more, the upper echelons theory posits that the TMT can form strategies via sharing information, resources, and decisions. Therefore, TMT information elaboration could influence innovative corporate decisions.

According to the above logic, TMT information elaboration, generated through CEO empowering leadership, motivates and facilitates employees’ innovative capabilities. In particular, the information elaboration among TMT members can facilitate unconventional and innovative thinking and enhance their problem-solving. The sharing of various kinds of knowledge, skills, and experiences among team members can inspire new ideas and knowledge. All of these can promote corporate entrepreneurship. Hence, we propose the following hypothesis:

*Hypothesis 3: TMT information elaboration mediates the relationship between CEO empowering leadership and corporate entrepreneurship.*

### The Moderating Effect of Environmental Dynamism on the Relationship Between Empowering Leadership and Information Elaboration

Environmental dynamism refers to the rate and unpredictability of environmental changes, such as product/service obsolescence, technological developments, competitor actions, and customer demand changes (Newkirk and Lederer, 2006). Managers working in more dynamic or rapidly changing environments face more risks. Highly dynamic environments are thus more likely to induce high levels of stress and worry among top team managers than less uncertain environments (Waldman et al., 2001). As a result, managers who are empowered by CEOs are likely to exert greater effort to collect and share information in order to reduce the potentially negative effects of environmental dynamism.

Top management team members in dynamic environments tend to rely on one another to obtain cues and information that will help them interpret the business situation and judge personal relationships. In addition, those responsible for the firm’s future development are more likely to generate new ideas and relish the opportunity to consider alternatives that reduce negative side effects, thus lessening the potential harm of the uncertain environment. In contrast, when the environment is stable, or at least less uncertain, TMT members who are delegated more power are less likely to realize that information elaboration is needed at the team level.

Therefore, we expect perceived environmental dynamism to have a positive moderating effect on the relationship between CEO empowering behavior and information elaboration in a TMT. When TMT members perceive themselves to be operating in a dynamic environment and to be empowered by their CEOs, they are more likely to share information with one another. This discussion brings us to our following hypothesis.

*Hypothesis 4: Environmental dynamism moderates the relationship between CEO empowering leadership and TMT information elaboration. All other conditions being equal, the higher the degree of environmental dynamism, the stronger the positive relationship between such empowering leadership and information elaboration.*

### The Moderating Effect of Environmental Dynamism on the Relationship Between Information Elaboration and Corporate Entrepreneurship

Although a number of studies have investigated the information elaboration-corporate entrepreneurship relationship (e.g., Waldman et al., 2001), few examine that relationship at the TMT level. Another major weakness of this body of research is its insufficient consideration of how contextual variables may moderate the relationship between information elaboration and corporate entrepreneurship. In this section, we discuss how one such variable, i.e., environmental dynamism, may moderate the relationship.

As noted, although research suggests the potential influence of information elaboration on corporate entrepreneurship, it remains unclear how environmental dynamism may influence the relationship between them. Zahra and George (2002) posit that corporate entrepreneurship can be influenced by the joint effect of environmental, organizational, and strategic factors. In an environment characterized by a high degree of dynamism or rapid change, organizational learning may take place more slowly, which can in turn affect the development of corporate entrepreneurship (Zahra and George, 2002). In addition, highly dynamic and rapidly changing environments may create tension over which entrepreneurial role each manager or TMT member should play, which may introduce strategic role conflict (Dess et al., 2003). Such role conflict may lead to difficulty in taking up entrepreneurial roles, thus exerting a negative effect on corporate entrepreneurship (Grover, 1993; Floyd and Lane, 2000). Ultimately, in a highly dynamic environment, too much information exchange/sharing may also slow down the decision-making process, making it more difficult for TMT members to reach consensus, which would also negatively affect the development of corporate entrepreneurship (Hirschman, 1970). For all of these reasons, we expect perceived environmental dynamism to have a negative moderating effect on the relationship between information elaboration and corporate entrepreneurship, and thus posit the following.

*Hypothesis 5: Environmental dynamism moderates the relationship between TMT information elaboration and corporate entrepreneurship. All other conditions being equal, the higher the degree of environmental dynamism, the weaker the positive relationship between information elaboration and corporate entrepreneurship.*

**FIGURE 1:**
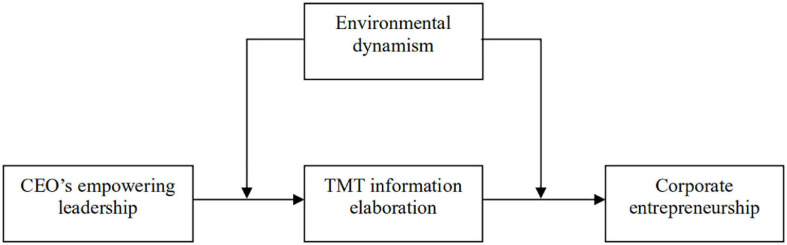
Conceptual model.

Based upon the foregoing hypotheses, the theoretical model is presented in Figure 1.

## Methodology

### Sample

The data of this study was collected from enterprises in mainland China. We mainly used two methods for distributing the questionnaires. The first method was randomly to visit enterprises in person and meet with their CEO. The second method was to collect data by questioning CEOs who were also EMBA students at a university. With the helping of these CEOs and EMBA students, we randomly selected companies and sent the invitation letter to illustrate this research purpose, together with a cover letter that guaranteed the anonymity and confidentiality of their responses. After contacting the CEOs and obtaining their approval, we requested that TMT members fill out and return the questionnaire. Questionnaires were then mailed or personally delivered to the firms. All participants were informed that participation was voluntary. Respondents were asked to return the completed questionnaire to the researchers in a prepaid return envelope. There were three different sets of surveys with one-month interval designed to obtain distinct perspectives on company operations, namely the perspectives of CEOs, TMT members (including other top managers, i.e., CHO and CFO), and CFOs. More specifically, CEOs answered questions related to environmental dynamism and TMT information elaboration; TMT members rated the CEO empowering leadership; and CFOs rated corporate entrepreneurship.

After removing incomplete questionnaires and matching up the sets, 149 useable matched responses were obtained from 422 TMT members and 149 CEOs from 149 firms. To reduce the possibility that corporate entrepreneurship was overly influenced by previous CEOs and not an outcome of the current CEO’s leadership, the analysis focused on firms in which CEOs had been in place for more than two years. Fifty-two organizations were excluded on this basis, which resulted in a final sample of 97 firms. Within the 97 firms, there were 97 CEOs, 235 TMT managers, and 97 CFOs who completed the questionnaires.

The final sample includes firms with various ownership types, including state-owned enterprises, foreign-invested enterprises, and other types such as private firms. Of the 97 firms, 32 (33.0%) are state-owned enterprises and 65 (67.0%) are non-state-owned enterprises (such as foreign-invested enterprises and private firms). These firms are located throughout China and include a variety of industries. Manufacturing firms account for 53.6% of the sample, with the remaining 46.4% of the sample being non-manufacturing firms.

### Measures

#### Empowering Leadership

A 15 item scale adapted from Arnold et al. (2000) was employed in this study for measuring CEO empowering leadership. To avoid any social desirability bias, the TMTs, rather than the CEOs, were asked to evaluate the extent to which the CEO empowers them, based on a 5-point Likert scale, ranging from 1 = “very low extent” to 5 = “very high extent.” Sample items included “Our CEO teaches our team members how to solve problems on our own,” and “Our CEO encourages us to speak out regarding our opinions and suggestions.” To read detailed mainscales used in this research, please see Appendix Table A1.

Since we measured empowering leadership by using the responses of TMT members, we utilized James et al.’s (1984) inter-rater reliability coefficient [*R*wg (j)] to justify aggregating the responses of individual members to the team level. An average value greater than or equal to 0.70 indicates good agreement within a group (James et al., 1984). According to our study results, the average Rwg value for empowering leadership is greater than 0.70, which justifies the aggregation of team members’ responses. The AMOS 7 software package was applied to perform a second-order confirmatory factor analysis (CFA) to assess the homogeneity of the five sub-dimensions. All the measurements were modeled to load to the corresponding sub-dimensions, and all five sub-dimensions were loaded to an overall higher order factor measuring empowering leadership. Convergent validity was examined by investigating the item loadings and their significance. The overall model’s chi-squared, comparative fit index (CFI; Chan, 1998), root mean square error of approximation (RMSEA; Bentler, 1990), and Tucker–Lewis Index (TLI; Browne and Cudeck, 1993) were used to assess model fit. The second-order CFA model fits the data very well (χ^2^ = 141.74, *df* = 85, TLI = 0.93, CFI = 0.94, RMSEA = 0.08). This means that these items were aggregated into a composite score for the subsequent analyses. The aggregate-level Cronbach’s reliability coefficient was calculated, and the alpha value was 0.94, indicating acceptable measurement reliability.

#### Elaboration of Information

Information elaboration was measured with a 4-item scale, which was developed by Kearney et al. (2009). The items, which were answered by CEOs, are based on a 5-point Likert scale, ranging from 1 = “very low extent” to 5 = “very high extent.” Sample questions included: “The members of this team complement each other by openly sharing their knowledge,” and “The members of this team carefully consider all perspectives in an effort to generate optimal solutions.” Cronbach’s reliability coefficient alpha value was 0.80.

#### Corporate Entrepreneurship

A 13-item scale developed by Zahra (1996) was adapted to measure corporate entrepreneurship. CFOs of the sample firms were asked to evaluate the extent of the firm’s engagement in entrepreneurial activities in the past, based on a 5-point Likert scale, ranging from 1 = “very low extent” to 5 = “very high extent.” Cronbach’s reliability coefficient alpha value was 0.88.

#### Environmental Dynamism

Environmental dynamism was measured by a 5-item scale developed by Jansen et al. (2006), which is also based on previous literature (Dill, 1958; Jansen et al., 2006). CEOs evaluated the extent of change and the instability of the external environment based on a 5-point Likert scale, ranging from 1 = “very low extent” to 5 = “very high extent.” The sample questions included: “Environmental changes in our local market are intense,” and “In our local market, changes are taking place continuously.” Cronbach’s reliability coefficient alpha value was 0.84.

#### Control Variables

We chose the following variables as control variables in the analysis. (1) Ownership structure: the reason for the inclusion of this control variable is that firms with different ownership structures may lead to different organizational performance (Volberda and Bruggen, 1997). We coded ownership structure as one for state owned, zero for non-state owned; (2) Industry type: we coded industry type as one for manufacturing firms, zero for non-manufacturing firms; (3) Firm age: firm age is a key factor influencing firms’ strategic choices and performance (Darnall and Edwards, 2006). A firm’s innovative strategies and performance vary with age. We thereby included firm age as a control variable in the analysis; (4) Firm size: we measured firm size as the number of full-time employees, as was done in previous research (Cardinal, 2001); (5) Environmental hostility: environmental hostility refers to the existence of unfavorable external forces in a firm’s business environment (Hannan and Freeman, 1984). In a hostile business environment, firms should consider each decision more extensively in order to face other important potential threats and opportunities, thus leading to different final performance. We also controlled for environmental hostility, with CEOs serving as the respondents for this measure. The five-point Likert-type scale was used, ranging from one (strongly disagree) to five (strongly agree). Cronbach’s alpha for this measure was 0.86, which is accepted.

### Statistical Analysis

We conducted three stages to analysis data. First, CFAs were conducted to test sufficient convergent and discriminant validity among all constructs. Second, descriptive statistics and correlation analysis were calculated to illustrate the interrelations among the study variables. Finally, multiple hierarchical regressions were employed to test the hypotheses as proposed above. Specifically, the relationship between empowering leadership (IV) and information elaboration (Mediator) was first tested, followed by the test on the relationship between empowering leadership (IV) and corporate entrepreneurship (DV). Following the usual practice for testing on the mediation (Zahra and Garvis, 2000), both IV and Mediator were put into the equation to observe their effects on DV. The moderating role of environmental dynamism was also tested by entering the interaction term of empowering leadership and environmental dynamism into the model. Then, to validate our results on the moderating role of environmental dynamism, we utilized bootstrapping methods to test the conditional indirect effects at different values of the moderator variable.

AMOS 24.0 was used for the CFAs to evaluate the discriminant validity of key variables, and SPSS 24.0 was used to calculate the descriptive statistics, correlation between variables, and multiple regression analyses. SPSS macro designed by Preacher et al. (2007) was used to test conditional indirect effects.

## Results

### Construct Validity

Confirmatory factor analyses were conducted to ensure sufficient convergent and discriminant validity among all constructs. Given the small sample size relative to the measurement items, we adopted procedures frequently used by researchers (Baron and Kenny, 1986). We reduced the number of items by creating three indicators for each single-dimension construct. Based on the factor analysis results, the items with the highest and lowest loadings for each construct were combined first, followed by the items with the next highest and lowest loadings, until all the items had been assigned to one of the indicators. Scores for each indicator were then computed as the mean of the scores on the items that constituted each indicator. We examined a four-factor CFA model that included empowering leadership, information elaboration, environmental dynamism, and corporate entrepreneurship. The proposed four-factor model fitted the data well [χ^2^ (71) = 102.66, *p* < 0.01; CFI = 0.96, TLI = 0.94; RMSEA = 0.07]. In addition, all of the loadings of indicators were significant at *p* < 0.01, with the standardized loadings ranging from 0.533 to 0.888, demonstrating convergent validity.

The discriminant validity of the four proposed constructs was tested by contrasting the four-factor model against alternative models. Model comparison results (see Table 1) revealed that the four-factor model fits the data considerably better than any of the alternative models. Thus, the distinctiveness of the four constructs in the study was supported. Given these results, all four variables were applied in the subsequent analyses.

**TABLE 1:** Results of confirmatory factor analysis for the measures of the variables studied.

*Model*	χ*^2^*	*df*	Δχ*^2^*	*TLI*	*CFI*	*RMSEA*
Four-factor model	102.66	71		0.94	0.96	0.068
Three-factor model 1: Empowering leadership and elaboration of information combined	135.24	74	32.58**	0.89	0.91	0.093
Three-factor model 2: Empowering leadership and environmental dynamism combined	199.80	74	97.14**	0.78	0.82	0.133
Three-factor model 3: Empowering leadership and corporate entrepreneurship combined	167.26	74	64.60**	0.84	0.87	0.115
Three-factor model 4: Elaboration of information and environmental dynamism combined	199.81	74	97.15**	0.78	0.82	0.133
Three-factor model 5: Elaboration of information and corporate entrepreneurship combined	166.96	74	64.30**	0.84	0.87	0.114
Three-factor model 6: Environmental dynamism and corporate entrepreneurship combined	171.01	74	68.35**	0.83	0.86	0.117
One-factor model	288.49	77	185.83**	0.64	0.70	0.169

*TLI, Tucker–Lewis index; CFI, comparative fit index; RMSEA, root-mean-square error of approximation.*

*p < 0.05; **p < 0.01.

### Descriptive Statistics

Table 2 presents the means, standard deviations, and correlations of all the variables. Empowering leadership is positively correlated with information elaboration and corporate entrepreneurship (*r* = 0.68 and 0.33; *p* ≤ 0.01, respectively). Information elaboration is positively correlated with corporate entrepreneurship (*r* = 0.33, *p* ≤ 0.01).

**TABLE 2:** Descriptive statistics.

Variables	Mean	SD	1	2	3	4	5	6	7	8
1. Empowering leadership	4.11	0.62	**(0.94)**							
2. Elaboration of information	3.91	0.67	0.68**	**(0.80)**						
3. Environmental dynamism	2.96	0.0.84	0.03	0.09	**(0.84)**					
4. Corporate entrepreneurship	3.44	0.60	0.33**	0.33**	0.24*	**(0.88)**				
5. Environmental hostility	3.07	0.82	0.02	0.03	0.75**	0.08	**(0.86)**			
6. Ownership structure^*a*^	0.33	0.47	0.18	0.20*	–0.04	–0.14	–0.08	–		
7. Industry type^*b*^	0.46	0.50	–0.19	−0.23*	0.03	–0.02	–0.04	–0.13	–	
8. Firm age	16.92	14.80	0.14	0.11	–0.07	–0.02	–0.14	0.36**	–0.02	–
9. Firm size^*c*^	1.79	0.81	0.25*	0.08	–0.09	0.10	–0.16	0.41**	–0.19	0.51^∗∗^

*Bold values in parentheses on the diagonal are the Cronbach’s alpha value of each scale.*

*^a^Coding: “state owned” = 1; “non-state owned” = 0.*

*^b^Coding: “manufacturing firm” = 1; “non-manufacturing firms” = 0.*

*^c^Coding: “small-sized firm” = 1; “medium-size firm” = 2; “large-size firm” = 3.*

**p ≤ 0.01; *p ≤ 0.05 (two-tailed).

### Tests of Hypotheses

Table 3 shows the results of the regression analyses used to test the hypotheses. The control variables, including ownership structure, industry type, firm age, firm size, and environmental hostility were entered first (Model 1), followed by Model 2. In the following Model 2, the relationship between empowering leadership and information elaboration was tested. Empowering leadership showed a positive association with information elaboration (β = 0.66, *p* < 0.01), and the R-squared change is significant. Thus hypothesis 2 was supported.

**TABLE 3:** Result of regression analysis^*a*^.

	Elaboration of information		Corporate entrepreneurship			Elaboration of information		Corporate entrepreneurship		
				
	M1	M2	M3	M4	M5	M6	M7	M8	M9	M10
**Control Variables**										
Ownership structure	0.17	0.09	–0.18	−0.22†	−0.24*	0.10	0.10	−0.24*	−0.22†	−0.20†
Industry type	–0.16	–0.07	0.02	0.02	0.04	–0.08	–0.07	0.04	0.00	0.02
Firm age	0.07	0.07	–0.04	–0.04	–0.06	0.06	0.07	–0.06	0.07	–0.10
Firm size	–0.04	–0.16	0.20	0.14	0.19	–0.17	–0.16	0.21	0.19	0.23†
Environmental hostility	0.01	–0.00	0.12	0.11	0.11	–0.11	–0.05	0.11	−0.20†	–0.19
**Independent Variables**										
Empowering leadership (EL)		0.66**		0.32**	0.13	0.65**	0.75**			
Elaboration of information (EOI)					0.29*			0.37**	0.34**	0.39**
**Moderator**										
Environmental dynamism (ED)						0.15	0.09		0.40*	0.41**
Interaction										
EL ^∗^ ED							0.23*			
EOI ^∗^ ED										−0.26*
*R* ^2^	0.07	0.46	0.06	0.15	0.20	0.47	0.51	0.19	0.25	0.31
Δ*R*^2^	0.07	0.39	0.06	0.09	0.05	0.01	0.04	0.14	0.06	0.06
*F*	1.08	10.29**	0.89	2.16*	2.49*	8.99**	9.13**	2.78**	3.35**	3.94**
Δ*F*	1.08	52.55**	0.89	8.07**	4.00*	1.11	5.89*	11.59**	5.70*	6.31*

*^a^Tabled value are standardized regression weights.*

**p < 0.01; *p < 0.05; ^†^p < 0.10 (two-tailed).

The relationship between empowering leadership and corporate entrepreneurship was tested in Model 4, which indicated that empowering leadership was positively associated with corporate entrepreneurship (β = 0.32, *p* ≤ 0.01). The change in R-squared was also significant, thus supporting hypothesis 1.

In Model 5, both empowering leadership and information elaboration were entered. Results indicated that after entering both empowering leadership and information elaboration into Model 5, the relationship between empowering leadership and corporate entrepreneurship became not significant, but the relationship between information elaboration and corporate entrepreneurship was significant. According to the mediation conditions suggested by Baron and Kenny (1986), information elaboration fully mediated the relationship between empowering leadership and corporate entrepreneurship, so hypothesis 3 was supported.

**FIGURE 2:**
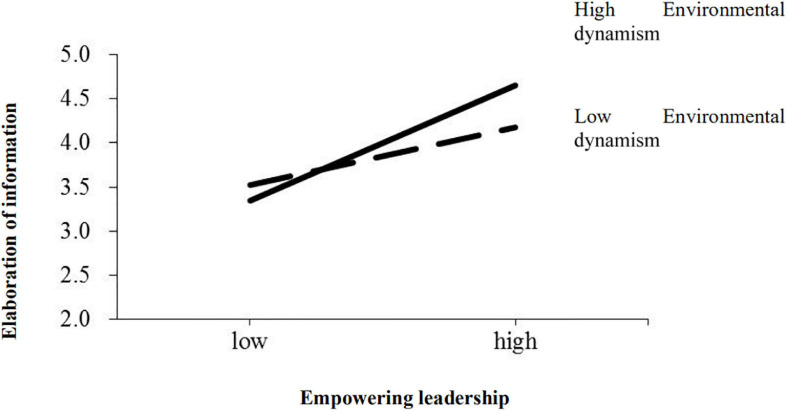
Interaction between empowering leadership and environmental dynamism.

The moderating effect of environmental dynamism on empowering leadership–elaboration of information linkage (hypothesis 4) was tested in Model 6 and Model 7. The moderator, environmental dynamism, was entered in Model 6 and the interaction of empowering leadership and environmental dynamism was added in Model 7. The R-squared changes of both models were significant, and the moderating effect of environmental dynamism was found to be significant in the empowering leadership–information elaboration relationship (β = 0.23, *p* < 0.05). The nature of the significant interaction of empowering leadership and environmental dynamism was examined by plotting figures with values plus and minus one standard deviation from the means of empowering leadership and environmental dynamism (Baron and Kenny, 1986). Figure 2 clearly illustrates the significant interactions. As shown in Figure 2, the relationship between empowering leadership and elaboration of information was stronger for firms operating in a high level of environmental dynamism than those operating in a low level of environmental dynamism. Therefore, environmental dynamism positively moderates the relationship between empowering leadership and information elaboration. Hypothesis 4 thus received support.

**FIGURE 3:**
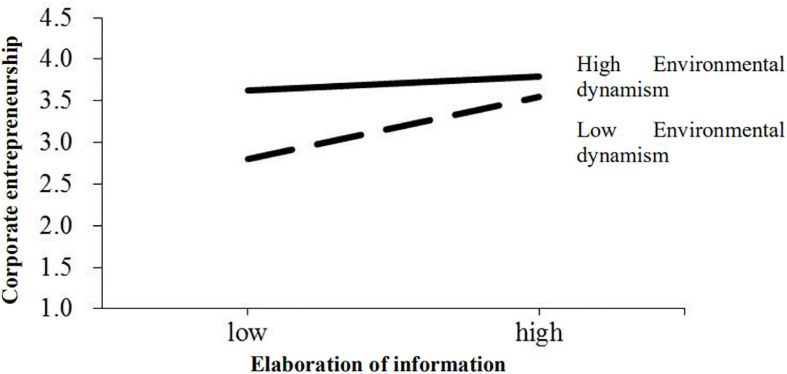
Interaction between elaboration of information and environmental dynamism.

Taking similar steps, the moderating effect of environmental dynamism on elaboration of information–corporate entrepreneurship linkage (hypothesis 5) was tested in Model 9 and Model 10. The moderator, environmental dynamism, was entered in Model 9 and the interaction of empowering leadership and environmental dynamism was added in Model 10. The R-squared changes of both models were significant, and the moderating effect of environmental dynamism was found to be significant in the elaboration of information–corporate entrepreneurship relationship (β = −0.26, *p* < 0.05). Figure 3 illustrates the significant interactions showing that the relationship between elaboration of information and corporate entrepreneurship was stronger for firms operating in a lower level of environmental dynamism than those operating in a higher level of environmental dynamism. Hypothesis 5 thus received support.

To validate our results for hypotheses 4 and 5, we utilized an SPSS macro designed by Preacher et al. (2007). This macro facilitates the implementation of the recommended bootstrapping methods and provides a method for probing the significance of conditional indirect effects at different values of the moderator variable. Table 4 presents the results for three of the hypotheses. Results indicated that the cross-product term between empowering leadership and environmental dynamism was significant (*B* = 0.27, *t* = 2.57, *p* < 0.05). Thus, hypothesis 4 was supported. Hypothesis 5 was also supported due to the cross-product term between elaboration of information and environmental dynamism being significant (*B* = −0.28, *t* = −2.11, *p* < 0.05). To fully support hypothesis 6, we examined the conditional indirect effect of empowering leadership on corporate entrepreneurship (through elaboration of information) at three values of environmental dynamism (see middle of Table 4): the mean (2.96), one standard deviation below the mean (2.12), and one standard deviation above the mean (3.79). Normal-theory tests indicated one of the three conditional indirect effects (based on moderator values at one standard deviation below the mean) was positive and significantly different from zero. Bootstrap confidence intervals corroborated these results. Preacher et al.’s (2007) macro also computes conditional indirect effects at various arbitrary values of the moderator that fall within the range of the data (see the lower half of Table 4). This output complements the more typical probing of the interaction using one standard deviation above and below the mean, and it allowed us to identify the values of environmental dynamism, for which the conditional indirect effect was just statistically significant at alpha = 0.05. Results demonstrated that the conditional indirect effect was significant at alpha = 0.05 for values of environmental dynamism at the intervals between 1.80 and 2.80.

**TABLE 4:** Regression results for conditional indirect effect.

Predictor	B	SE	*t*-value	*p*-value
**Elaboration of information**				
Constant	3.76	1.25	3.01	0.00
Empowering leadership	0.02	0.29	0.06	0.95
Environmental dynamism	–1.07	0.44	–2.43	0.02
Empowering leadership × environmental dynamism	0.27	0.10	2.57	0.01
**Corporate entrepreneurship**				
Constant	–0.71	1.63	–0.44	0.66
Empowering leadership	–0.10	0.40	–0.24	0.81
Environmental dynamism	0.89	0.55	1.60	0.11
Empowering leadership × environmental dynamism	0.09	0.14	0.64	0.52
Elaboration of information	1.03	0.43	2.41	0.02
Elaboration of information × environmental dynamism	–0.28	0.13	–2.11	0.04

**Environmental dynamism**	**Boot indirect effect**	**Boot SE**	**Boot *z***	**Boot *p***

**Conditional indirect effect at environmental dynamism = Mean ± 1 SD**				
−1 SD (2.12)	0.26	0.12	2.14	0.03
Mean (2.96)	0.17	0.09	1.86	0.06
+1 SD (3.79)	–0.02	0.13	–0.17	0.87

**Environmental dynamism^*a*^**	**Boot indirect effect**	**Boot SE**	**Boot *z***	**Boot *p***

**Conditional indirect effect at range of values of environmental dynamism**				
1.000	0.21	0.18	1.18	0.24
1.200	0.23	0.16	1.44	0.15
1.400	0.25	0.15	1.69	0.09
1.600	0.26	0.14	1.90	0.06
1.800	0.26	0.13	2.08	0.04
2.000	0.26	0.12	2.21	0.03
2.200	0.25	0.11	2.27	0.02
2.400	0.24	0.11	2.27	0.02
2.600	0.22	0.10	2.17	0.03
2.800	0.19	0.10	1.97	0.05
3.000	0.16	0.10	1.63	0.10
3.200	0.12	0.10	1.18	0.24
3.400	0.08	0.11	0.70	0.49
3.600	0.03	0.13	0.23	0.82
3.800	–0.03	0.15	–0.17	0.86
4.000	–0.09	0.18	–0.49	0.63
4.200	–0.16	0.21	–0.73	0.46
4.400	–0.23	0.25	–0.92	0.36
4.600	–0.31	0.29	–1.06	0.29
4.800	–0.39	0.33	–1.17	0.24
5.000	–0.48	0.38	–1.26	0.21

*N = 97. Unstandardized regression coefficients are reported. Bootstrap sample size = 5000.*

*^a^Range of values represent an abbreviated version of the output provided by the macro.*

## Discussion

This study examines the proposition that CEO empowering leadership facilitates information elaboration among TMT members, which in turn results in positive effects on corporate entrepreneurship. The findings provide empirical support for this proposition. We found that TMT information elaboration fully mediates the relationship between CEO empowering leadership and corporate entrepreneurship and that environmental dynamism positively moderates the relationship between empowering leadership and information elaboration but negatively moderates the relationship between information elaboration and corporate entrepreneurship. We have found that a stronger positive relationship between CEO empowering leadership and information elaboration and a weaker positive relationship between information elaboration and corporate entrepreneurship can be observed for firms operating in a highly dynamic environment.

### Theoretical Implications

This study has a number of important theoretical implications that suggest direction for future research. Firstly, these findings contribute to the current understanding of the empowering leadership-performance linkage by relating it to senior executives such as CEOs (e.g., Preacher et al., 2007). The roles of CEOs might be very different from those of lower-level team leaders. For example, lower-ranked team leaders usually focus on a set of non-strategic specific team tasks. However, CEOs should deal with many complex and uncertain problems (Hambrick, 1994; Carmeli et al., 2011) and more strategic decisions important to the survival and success of their organization. To fully achieve their objectives, CEOs should integrate their efforts with TMT members, who are in charge of different departments (Ling et al., 2008). The influence CEOs have on their direct subordinates (TMT members) is of great significance to a firm’s performance. Therefore, this study adds further understanding of the CEO empowering leadership-performance relationship by paying attention to empowering CEOs and examining their impacts on corporate entrepreneurship, which also takes into consideration the moderating role of environmental dynamism.

Secondly, the evidence for TMT information elaboration as a mediator helps elucidate the mechanism by which CEO empowering leadership improves firm performance. The mechanism of TMT information elaboration has not been previously studied as the process through which empowering leadership can impact firm performance. Our findings suggest a new role for CEOs, i.e., facilitator of information elaboration. Although prior research has tested other CEO roles in the TMT context (e.g., Hambrick, 1994), the information elaboration facilitator role has been insufficiently investigated. It should also be pointed out that the roles of leaders at the TMT level can be very different from those of lower-level team leaders. For example, lower-level team leaders usually focus on a set of specific non-strategic team tasks, whereas CEOs deal with many complex and uncertain problems (Hambrick, 1994; Carmeli et al., 2011) that affect strategic decisions. Because of these differences, it would be helpful to study the effect of information elaboration among CEOs and their teams (Ling et al., 2008). Our data support the organizational learning perspective on corporate entrepreneurship proposed by several authors (e.g., Hambrick, 1994; Dess et al., 2003), which highlights the importance of information exchange and elaboration on the development of such entrepreneurship, shedding new light on and suggesting the complexity of this issue. Thus, these findings significantly extend the existing research (e.g., Vecchio et al., 2010; Wei and Wu, 2013), which studied the ways CEOs facilitate TMT processes, boosting firm performance. The results of this study support the argument that CEOs’ empowerment of TMT members can enhance their motivation to share and exchange information. With the increasing elaboration and integration of information, TMT members are able to consider unconventional and innovative ways or solutions to deal with their work, which is beneficial to the firm’s corporate entrepreneurship. It is the information elaboration that helps improve the firm’s overall entrepreneurial activities.

Thirdly, owing to the effect of environmental factors such as environmental dynamism, the processes by which empowering leadership affects TMT information elaboration and the ways in which information elaboration improves corporate entrepreneurship are more complicated than prior studies have suggested. That is, the positive relationships above may actually change in the face of a high level of environmental dynamism. In other words, the relationships between empowering leadership and TMT information elaboration and information elaboration and corporate entrepreneurship should be contingent upon environmental factors. In particular, prior research has paid insufficient attention to the moderating effect of such factors on the relationship between information elaboration and corporate entrepreneurship. Even in those well-written summaries on the relationship between organizational learning and information exchange on the one hand, and the development of corporate entrepreneurship on the other (e.g., Dess et al., 2003), the moderating effects of environmental variables are not adequately considered. Therefore, our results suggest a new direction for conducting future studies to understand the relationship between information elaboration and the development of corporate entrepreneurship, i.e., conducting more research on the direct and moderating effects of contextual variables. Further research in this direction should be able to provide theory-supported answers to such questions as why the seeming lack of information elaboration in many entrepreneurial firms (e.g., Apple and many Asian high-tech firms) does not prevent the firms from developing corporate entrepreneurship. According to the results of our current study, it is the environmental factor that may hold the answer. Therefore, more research considering the effects of these variables will theoretically help enhance the corporate entrepreneurship literature.

### Practical Implications

The findings of this study also have several managerial implications for business practitioners. Firstly, we found significant effects of CEO empowering leadership on corporate entrepreneurship. This confirms the importance of leadership style for improving a firm’s overall entrepreneurial activities. The CEO is an important figure in an organization, especially in the context of China. In traditional Chinese values, leaders have supreme authority in organizations (Carmeli et al., 2011). CEOs are the most powerful individuals in decision making, and their leadership style, such as empowerment, can influence TMT behaviors and thus organizational performance (Hambrick, 2007; Wang et al., 2011). The findings showed that TMT members play a significant role in the CEO’s decision-making process. Therefore, when focusing on the specific context of business in China, this study should be of great importance in understanding the influence of a CEO’s leadership behaviors and their relation to firm performance.

Secondly, we found that information elaboration acts as a mediator linking empowering leadership to corporate entrepreneurship. As a result, we obtained evidence showing that empowering leadership helps to improve information elaboration, and information elaboration can improve the development of corporate entrepreneurship, which is particularly helpful to CEOs in East Asian firms that may be lacking in this regard. CEOs should therefore place a greater focus on fostering information exchange and integration in TMTs. For example, TMT members experiencing empowerment from their CEO will find their work more meaningful and stimulating. They will feel a greater sense of autonomy and freedom to plan and conduct their work, which can foster intrinsic motivation for information elaboration and achieve enhanced corporate entrepreneurship. This information elaboration, nevertheless, is a result of effective empowerment by CEOs. Thus, firms may need to focus more on how to develop an empowering leadership style in their CEOs in order to foster the motivation of TMT members to share information and be more innovative. Research shows that corporate entrepreneurship helps to improve firm performance (e.g., Hambrick et al., 2005), and our findings suggest that greater information elaboration may help to improve such performance by generating more corporate entrepreneurship.

Thirdly, our results suggest that environmental dynamism positively moderates the relationship between empowering leadership and information elaboration. Accordingly, less empowering leadership should also reduce the positive interactive effect on information elaboration. In addition, we also obtained evidence to show that environmental dynamism moderates the relationship between information elaboration and corporate entrepreneurship in a negative direction, which suggests that it may be unhelpful for CEOs to overly encourage information elaboration in highly uncertain environments. Too much information and information exchange may impact the effectiveness and efficiency of decision-making. When considering the role of environmental dynamism in the process of information elaboration, it is necessary for firms to pay greater attention to environmental factors in order to lessen the potential harm that may result from environmental uncertainty.

### Limitations and Future Research

This study had several limitations that also suggest direction for future research. One such limitation is that the study was not experimental in nature, and thus no causal effect could be fully confirmed. Moreover, we limited our examination of the mediating effect of information elaboration on the relationship between empowering leadership and corporate entrepreneurship, but there may be other mediators that influence the relationship above. A more comprehensive study comparing the relative importance of information elaboration and other mediators to the development of corporate entrepreneurship is recommended in order to afford a better understanding of the mediating effect of information elaboration.

In addition, as noted, our sample size was relatively small and comprised Chinese firms alone, which weakens the predictive validity of our data and the generalizability of our research findings (Dess et al., 2003). Future studies in this arena should ensure a larger sample size and collect data from other countries or cultures.

Despite these limitations, our study still has significant strengths. For example, we collected data from three different sources: we asked CEOs to answer questions related to environmental dynamism and TMT information elaboration, TMT members to rate the degree to which their leaders (CEOs) empower them, and CFOs to provide information about the degree of corporate entrepreneurship in their firms. Such a diversity of informants gives us greater confidence in the strength of our conclusions. All of the interesting new findings outlined herein will help scholars to better understand the complex relationships between both empowering leadership and corporate entrepreneurship.

## Conclusion

The findings of this study contribute to the corporate entrepreneurship research by linking it to empowering leadership and information elaboration. Prior research has considered the effects of empowering leadership and information elaboration at the ordinary group level alone, whereas our study considered these effects at the TMT level, thereby furthering our understanding of the relationship among empowering leadership, information elaboration, and corporate entrepreneurship. Our findings suggest that information elaboration is less likely to improve corporate entrepreneurship in a highly uncertain environment, although empowering leadership can enhance such elaboration in this type of environment. Future research should further explore the effects of information elaboration on different dimensions of firm performance and consider the influence of more environmental or contextual factors.

## Data Availability Statement

The original contributions presented in the study are included in the article/supplementary material, further inquiries can be directed to the corresponding author/s.

## Author Contributions

ZL was responsible for the writing of the main research ideas, research questions, and article structure of this manuscript. HC was mainly responsible for data analysis and conclusions. QM was responsible for collecting and collating documents, adjusting the format of the article, and the submitting work. HL was mainly responsible for the investigation of preliminary research issues and data collection. All authors contributed to the article and approved the submitted version.

## Conflict of Interest

The authors declare that the research was conducted in the absence of any commercial or financial relationships that could be construed as a potential conflict of interest. The reviewer, SZ, declared a shared affiliation with one of the authors, ZL, to the handling editor at the time of review.

## Appendix

**TABLE A1:** Main scales used in this research.

Empowering leadership	Please indicate to what extent you agree/disagree with the following statements. (1 = strongly disagree to 5 = strongly agree)
	1: Our leader sets high standards for performance by his/her own behavior;
	2: Our leader works as hard as anyone in my work group;
	3: Our leader sets a good example by the way he/she behaves;
	4: Our leader encourages work group members to express ideas/suggestions;
	5: Our leader gives all work members a chance to voice their opinions;
	6: Our leader makes decisions that are based only on his/her own ideas;
	7: Our leader helps my work group see areas in which we need more training;
	8: Our leader teaches work group members how to solve problems on their own;
	9: Our leader encourages work group members to solve problems together;
	10: Our leader explains company decisions;
	11: Our leader explains the purpose of the company’s policies to my work group;
	12: Our leader explains his/her decisions and actions to my work group;
	13: Our leader cares about work group members’ personal problems;
	14: Our leader gets along with my work group members;
	15: Our leader gives work group members honest and fair answers.
**Corporate entrepreneurship**	**Please indicate to what extent you agree/disagree with the following statements. (1 = strongly disagree to 5 = strongly agree)**
	1: Our firm is spending heavily (well above the industry average) on product development;
	2: Our firm is introducing a large number of new products to the market;
	3: Our firm is acquiring significantly more patents than its major competitors;
	4: Our firm is pioneering the development of breakthrough innovations in its industry;
	5: Our firm is spending on new product development initiatives;
	6: Our firm is entering new markets;
	7: Our firm is establishing or sponsoring new ventures;
	8: Our firm is finding new niches in current markets;
	9: Our firm is changing its competitive approach (strategy) for each business unit;
	10: Our firm is reorganizing operations, units, and divisions to ensure increased coordination and communication among business units;
	11: Our firm is redefining the industries in which it competes;
	12: Our firm is introducing innovative HRM programs;
	13: Our firm is first in the industry to introduce new business concepts and practices.
**Information elaboration**	**Please indicate to what extent you agree/disagree with the following statements. (1 = strongly disagree to 5 = strongly agree)**
	1: The members of this team complement each other by openly sharing their knowledge;
	2: The members of this team carefully consider all perspectives in an effort to generate optimal solutions;
	3: The members of this team carefully consider the unique information provided by each individual team member;
	4: As a team, we generate ideas and solutions that are much better than those we could develop as individuals.
**Environmental dynamism**	**Please indicate to what extent you agree/disagree with the following statements. (1 = strongly disagree to 5 = strongly agree)**
	1: The environment is very dynamic, changing rapidly in technical, economic, and cultural dimensions;
	2: The environment is very risky and one false step can mean the firm’s undoing;
	3: The environment is very rapidly expanding through the expansion of old markets and the emergence of new ones;
	4: The environment is very stressful, exacting, hostile, hard to keep afloat.
