# Corrigendum: CEO Empowering Leadership and Corporate Entrepreneurship: The Roles of TMT Information Elaboration and Environmental Dynamism

**DOI:** 10.3389/fpsyg.2021.778349

**Published:** 2021-10-28

**Authors:** Zhengfei Li, Huangen Chen, Qiuying Ma, Haibo Li

**Affiliations:** ^1^School of Economics, Shandong University, Jinan, China; ^2^School of Business Administration, Faculty of Business Administration, Southwestern University of Finance and Economics, Chengdu, China; ^3^Department of Economics, University of Warwick, Coventry, United Kingdom; ^4^Institute of Science and Technology for Development of Shandong, Qilu University of Technology (Shandong Academy of Sciences), Jinan, China

**Keywords:** empowering leadership, information elaboration, corporate entrepreneurship, environmental dynamism, top management teams

In the published article, there were errors in affiliations 1 and 4. For affiliation 1, instead of “School of Management, Shandong University, Jinan, China”, it should be “School of Economics, Shandong University, Jinan, China.” For affiliation 4, instead of “Institute of Science and Technology for Development of Shandong, Qilu University of Technology, Shandong Academy of Science, Jinan, China”, it should be “Institute of Science and Technology for Development of Shandong, Qilu University of Technology (Shandong Academy of Sciences), Jinan, China.”

The authors apologize for these errors and state that they do not change the scientific conclusions of the article in any way.

The original article has been updated.

## Publisher's Note

All claims expressed in this article are solely those of the authors and do not necessarily represent those of their affiliated organizations, or those of the publisher, the editors and the reviewers. Any product that may be evaluated in this article, or claim that may be made by its manufacturer, is not guaranteed or endorsed by the publisher.

